# Supplementary material for: Blood Flow Restriction Is Not Useful as Soccer Competition Recovery in Youth Male National-Level Soccer Players: A Crossover Randomised Controlled Trial
Source: Sports (Basel). 2023 May 7;11(5):99. doi: 10.3390/sports11050099 (PMC10223773; doi:10.3390/sports11050099)
Supplement: Supplementary file 1 [file sports-11-00099-s001.zip › Table S1.pdf]

Table S1. Results from wellness questionnaire items, according to BFR or NoBFR condition as recovery session.

Wellness items (1 to 5 score)	BFR Mean (SD)	NoBFR Mean (SD)
**Fatigue**		
Match day	4.15 (0.86)	4.15 (0.87)
Post24h	2.87 (0.85)	3.07 (0.92)
Post48h	3.67 (0.76)	3.89 (0.85)
Post72h	4.20 (0.79)	4.20 (0.76)
**Sleep**		
Match day	4.60 (0.54)	4.56 (0.64)
Post24h	4.12 (0.82)	4.05 (1.07)
Post48h	4.35 (0.76)	4.33 (0.80)
Post72h	4.45 (0.67)	4.28 (0.82)
**Muscle soreness**		
Match day	3.95 (0.93)	3.87 (0.89)
Post24h	2.87 (0.93)	3.07 (1.01)
Post48h	3.50 (0.78)	3.74 (0.90)
Post72h	3.97 (0.80)	4.12 (0.86)
**Stress**		
Match day	4.20 (0.99)	4.15 (0.90)
Post24h	4.05 (0.98)	4.12 (0.89)
Post48h	4.25 (0.98)	4.15 (0.90)
Post72h	4.05 (1.06)	4.28 (0.82)
**Mood**		
Match day	4.40 (0.70)	4.30 (0.73)
Post24h	4.22 (0.79)	4.23 (0.80)
Post48h	4.27 (0.78)	4.20 (0.65)
Post72h	4.32 (0.65)	4.20 (0.95)

BFR, blood flow restriction; SD, standard deviation
