# Supplementary figures and images for: Blood Flow Restriction Is Not Useful as Soccer Competition Recovery in Youth Male National-Level Soccer Players: A Crossover Randomised Controlled Trial
Source: Sports (Basel). 2023 May 7;11(5):99. doi: 10.3390/sports11050099 (PMC10223773; doi:10.3390/sports11050099)

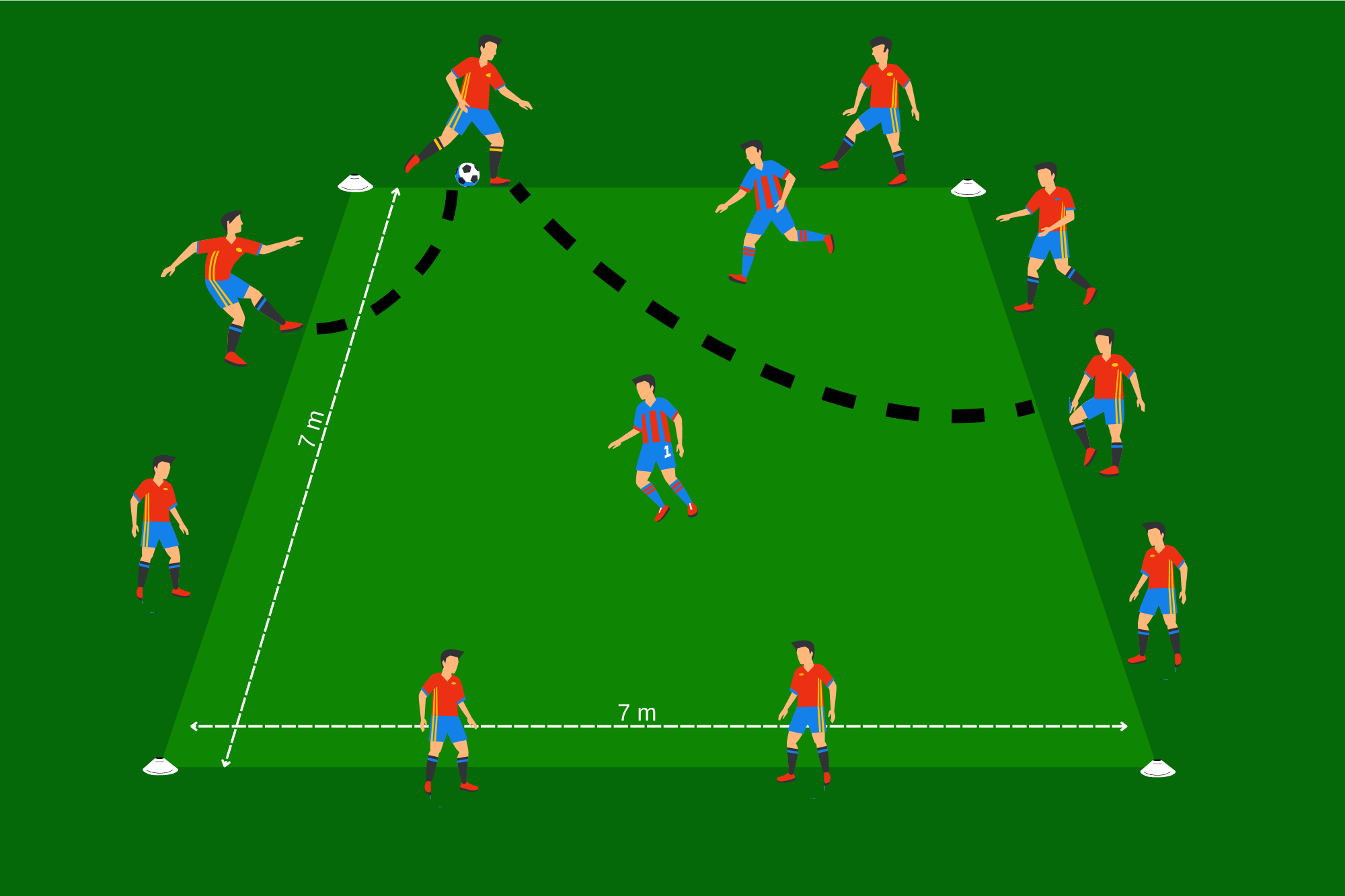

Supplement: Supplementary file 1 [file sports-11-00099-s001.zip › Figure S1.png]
